# The development and validation of the advance care planning questionnaire in Malaysia

**DOI:** 10.1186/s12910-016-0147-8

**Published:** 2016-10-18

**Authors:** Pauline Siew Mei Lai, Salinah Mohd Mudri, Karuthan Chinna, Sajaratulnisah Othman

**Affiliations:** 1 Department of Primary Care Medicine, University of Malaya Primary Care Research Group, Faculty of Medicine, University of Malaya, Kuala Lumpur, Malaysia; 2 Department of Social Preventive Medicine, Julius Centre, Faculty of Medicine, University of Malaya, Kuala Lumpur, Malaysia

**Keywords:** Advance care planning, Advance directives, Care plan, End of life care, Elderly, Validation, Malaysia

## Abstract

**Background:**

Advance care planning is a voluntary process whereby individual preferences, values and beliefs are used to aid a person in planning for end-of-life care. Currently, there is no local instrument to assess an individual’s awareness and attitude towards advance care planning. This study aimed to develop an Advance Care Planning Questionnaire and to determine its validity and reliability among older people in Malaysia.

**Methods:**

The Advance Care Planning Questionnaire was developed based on literature review. Face and content validity was verified by an expert panel, and piloted among 15 participants. Our study was conducted from October 2013 to February 2014, at an urban primary care clinic in Malaysia. Included were those aged >50 years, who could understand English. A retest was conducted 2 weeks after the first administration.

**Results:**

Participants from the pilot study did not encounter any problems in answering the Advance Care Planning Questionnaire. Hence, no further modifications were made. Flesch reading ease was 71. The final version of the Advance Care Planning Questionnaire consists of 66 items: 30 items were measured on a nominal scale, whilst 36 items were measured on a Likert-like scale; of which we were only able to validate 22 items, as the remaining 14 items were descriptive in nature. A total of 245 eligible participants were approached; of which 230 agreed to participate (response rate = 93.9 %). Factor analysis on the 22 items measured on a Likert-scale revealed four domains: “feelings regarding advance care planning”, “justifications for advance care planning”, “justifications for not having advance care planning: fate and religion”, and “justifications for not having advance care planning: avoid thinking about death”. The Cronbach’s alpha values for items each domain ranged from 0.637–0.915. In test-retest, kappa values ranged from 0.738–0.947.

**Conclusions:**

The final Advance Care Planning Questionnaire consisted of 63 items and 4 domains. It was found to be a valid and reliable instrument to assess the awareness and attitude of older people in Malaysia towards advance care planning.

**Electronic supplementary material:**

The online version of this article (doi:10.1186/s12910-016-0147-8) contains supplementary material, which is available to authorized users.

## Background

Advance care planning (ACP) is a process that expresses the preferences of an individual via verbal or written communications, for future health and personal care, and helps prepare people for healthcare decision-making in times of medical crisis [1]. The ACP process usually results in the designation of a health care proxy. ACP requires communication between patients, their family, and their health care providers, and reflects the patient’s relationships, culture, goals, values, and wishes about future healthcare, which will then drive specific medical treatment decisions that can be recorded in an advance directive [2]. ACP is now recognized to be more than a way to increase advance directives completion [3].

ACP is one of the several initiatives taken in developed countries in view of an aging population. It aims at improving the quality of end-of-life care among the elderly [4]. Since the 1990’s, ACPs have been widely promoted and supported by law in developed countries such as the United States, Australia, New Zealand and Canada [1, 5]. The process of ACP informs and empowers patients to have a say about their current and future treatment [6], and promotes the wishes of patients to die with dignity and free of pain, when further treatment and procedure show no benefit [7]. Those who have gone through ACP have shown an increased sense of control, hope and perception that their relationships with others have grown stronger [8].

In the United States, 30 % of Medicare expenditures are due to 5 % of those who die each year [9]. Approximately one-third of the expenditures in the last year of life is spent in the last month, from life-sustaining care [10]. Well implemented ACP policies have shown a reduction in cost for terminally ill patients [10]. In addition, family members of those that had ACP had less stress, less anxiety, less depression and higher satisfaction compared to those who received usual medical care [6]. Unfortunately, despite having good support for ACP in developed countries, the uptake of ACP is still low [4, 11].

In Japan, a survey administered to 560 residents in Tokyo (mean age = 44.7 ± 14.2 years), where 90 % of respondents rated their health status as good or fairly good, found that of the 156 that responded to know about living wills, only 12(7.7 %) had actually written one out [12]. In Hong Kong, a survey administered to 219 elderly patients (mean age = 73 ± 8 years) found that 81 % have never heard about ACP, and 73 % have never discussed this issue with others [13]. In Singapore, a similar survey was conducted among 414 residents (aged 21–100 years), only 37.9 % participants reported that they knew about ACP. Participants who did not wish “to be kept indefinitely on a life-support machine” and “accepted the “imminence of death” were found to have the willingness to sign a living will [4]. In Malaysia, a qualitative study performed among 15 elderly (aged 65–83 years) found that they have never heard of ACP or its concept [14]. When asked on whether they had any thoughts about their future illness, the majority revealed that they had not given it any thought, and that it was best to leave their future to fate or God [14]. However, these participants reported that they were open to the concept of ACP.

To date, there is no legislation that supports ACP in Malaysia. When a patient in Malaysia reaches a critical period where a major decision needs to be made as to whether active medical intervention needs to be provided to prolong life, the decision falls onto the doctors, or the next of kin. This causes a lot of stress to the decision maker(s), as they are not aware of the patients’ wishes. Currently, there is no information on the attitudes of older people residing in Malaysia regarding ACP. Thus, the aim of our study was to develop and validate the Advance Care Planning Questionnaire (ACPQ) in Malaysia, so that it can be used to assess the awareness and attitude of older people in Malaysia regarding ACP.

## Methods

### Development of the advance care planning questionnaire

The ACPQ was developed based on literature search in Ovid, PubMed and SAGE; using key words such as “advance care planning”, “advance directives”, “advance medical directive”, “care plan”, “end-of-life decision making”, “decision making”, “elderly”, “aging population”, “older people”. Language was limited to English. In the search, four relevant Asian studies were identified that were relevant to the Asian context [4, 12–14]. However, consent to use the instruments/research findings were only obtained from two studies [12, 14]. In assessing participants’ awareness, preferences, attitude and behaviour towards ACP, all 13 items from the Japanese questionnaire [12] were adopted. An additional 10 items were formulated based on a Malaysian qualitative study [14]. We decided to develop the ACPQ in English, as English is an important second language and is widely spoken among the elderly in countries which were ex-colonies of the United Kingdom, such as Malaysia [15].

The ACPQ consists of 66 items: 30 items were measured on a nominal scale, whilst 36 items were measured on a Likert-like scale (which focused on wishes in case of future incapacity, life-sustaining treatments, and the process of ACP as a whole) (Table 1 and Additional file 1: Appendix 1). However, we were not able to validate 14 items [“Topics they would like to discuss during ACP” (10 items), and “How you would like to record your ACP wishes” (4 items)] as the responses obtained were descriptive in nature. Hence, only 22 items were validated.

**Table 1:** The initial version of the Advance Care Planning Questionnaire

Section	Description	No. of items	Domain	Type of data
A	Demographic information	9	NA	Nominal scale
B	Health information	2	NA	Nominal scale
C	Knowledge on advance care planning	9	Knowledge	Nominal scale
D	Issues regarding advance care planning	2	Awareness	Nominal scale
		5	Previous experiences in the last 5 years	Nominal scale
		5	Feelings regarding advance care planning^a^	5 point likert-like scale (strongly agree to strongly disagree)
		7	Justifications for advance care planning^a^	5 point likert-like scale (strongly agree to strongly disagree)
		10	Justifications for not having an advance care plan^a^	5 point likert-like scale (strongly agree to strongly disagree)
		2	Intention to plan their advance care planning in advance	Nominal scale
		10	Topics they would like to discuss during advance care planning	5 point likert-like scale (strongly agree to strongly disagree)^b^
		4	How you would like to record your advance care planning wishes	5 point likert-like scale (strongly agree to strongly disagree)^b^
		1	Person to appoint as a decision maker	Nominal
	Total	66		

^a^Only items in these domains were tested for construct validity

^b^Although the response provided were of a 5 point likert-like scale, items in these domains were not tested for construct validity as the responses provided were descriptive

*NA* Not applicable

Face and content validity of the 66 items was verified by an expert panel which consisted of six academicians (two palliative care physicians, two geriatricians, one family medicine specialist and one family medicine clinical postgraduate candidate). Each item was reviewed, and the relevance and appropriateness of each item was discussed. Some items were deleted, some were rephrased and some new items were added, until the expert panel deemed that the ACPQ covered all the important domains on ACP. We hypothesized that the ACPQ would consist of three domains: “feelings regarding ACP”, “justifications for ACP”, and “justifications for not having ACP”. All participants were required to answer the 5 items in the “feelings regarding ACP” domain. Those who were in favour of ACP were then required to answer the 7 items in the “justifications for ACP” domain. For those who were not in favour of ACP, they were required to answer the 10 items in the “justifications for not having ACP” domain.

### Flesch reading ease

Flesch reading ease was calculated to assess the reading comprehension level of the ACPQ. This was calculated based on the average number of syllables per word and words per sentence. The higher the score, the easier it is to understand the document. An average document should have a score between 60–70 [16].

A pilot test was conducted on 15 participants aged more than 50 years. A researcher assisted the participants to answer the ACPQ as the questionnaire contained some medical terms that a lay person may not understand. They were asked to evaluate verbally if any of the items were difficult for them to understand. The time taken to complete the questionnaire ranged from 15–20 min.

### Validation of the advance care planning questionnaire

#### Study design and setting

This validation study was conducted from October 2013 to February 2014 at an urban primary care clinic in Malaysia.

### Participants

Patients who presented for their follow up at our primary clinic during the study period, who fulfilled our inclusion criteria (aged 50 years and above, and who could answer the questionnaire in English) were invited to participate in our study. Excluded were those with dementia, psychosis or those who were mentally challenged.

### Sample size

Sample size was calculated based on a rule of thumb of 10 participants per item in the ACPQ to perform factor analysis [17]. There were 22 items that were measured using a Likert scale in our instrument, that could be validated. Hence, the number of participants required was 22*10 = 220.

### Procedure

Eligible participants were approached after they had registered at the triage to see a doctor. After written informed consent was obtained, the ACPQ was administered by the researcher via a face-to-face interview. The time taken to complete the questionnaire ranged from 15–20 min. Retest was performed 2 weeks later.

Ethics approval was obtained from the University Malaya Medical Centre Medical Ethics Committee (MEC reference no: 938.16) prior to the study.

### Data analyses

All analyses were performed using the Statistical Package for Social Sciences version 22.0 (Chicago, Illinois, USA).

### Validity

Exploratory factor analysis was used as a data reduction technique, to look into the dimensionality of the ACPQ. In factor analysis, inter-item correlation, the Keiser-Meir-Olkin (KMO) value, the number of factors and the factor loadings were observed. The average variance extracted (AVE) and the composite reliability (CR) values were computed based on the factor loadings. KMO, factor loadings, AVE and CR values of more than 0.7, 0.5, 0.5 and 0.6, respectively, indicate good construct validity [18].

### Reliability

In reliability analysis, Cronbach’s α was used to assess the internal consistency of the items in ACPQ. This was calculated for the 22 items measured on Likert-like scale, as well as for each domain. Cronbach alpha values more 0.9 suggest redundancy of some items, values 0.70–0.90 imply adequate internal consistency, values 0.50–0.69 indicate poor internal consistency, and values below 0.50 indicate unacceptable internal consistency [19]. Corrected item-total correlations were then used to identify items which did not agree well with other items in the questionnaire. Corrected item-total correlation values should exceed 0.2 to be considered as acceptable [19].

Test-retest reliability was assessed using Cohen’s kappa coefficient. Kappa values can range from −1 to +1. Negative values are observed when the agreement is less than that expected by chance, and +1 shows complete agreement. Kappa values can be interpreted as follows: <0 less than chance agreement, 0.01–0.20 slight agreement, 0.21–0.40 fair agreement, 0.41–0.60 moderate agreement, 0.61–0.80 substantial agreement and 0.81–1.00 almost perfect agreement [20].

## Results

### Development of the Advance Care Planning Questionnaire

#### Flesch Reading ease

No problems were reported in the pilot study. Hence, no further changes were made to the ACPQ. Flesch reading ease of the ACPQ was 71.

### Validation of the Advance Care Planning Questionnaire

A total of 245 eligible participants were approached; out of which 230 agreed to participate (response rate = 93.9 %). The demographic characteristics and health status of participants are shown in Table 2.

**Table 2:** Demographic and health status of participants

	N (%) (*n* = 230)
Mean age ± SD (years) [range]	68.2 ± 6.8 [50–90]
Female	117 (50.9)
Ethnicity	
Chinese	99 (43.0)
Malay	71 (30.9)
Indian	60 (26.1)
Marital status	
Single	10 (4.3)
Married	177 (77.0)
Divorced	3 (1.3)
Widowed	40 (17.4)
Level of education	
None	1 (0.4)
Primary (completed 6 years of education)	24 (10.4)
Secondary (completed 12 years of education)	126 (54.8)
Tertiary (completed at least 15 years of education)	79 (34.3)
Currently working	19 (8.3)
Religion	
Islam	73 (31.7)
Buddhism	59 (25.7)
Christian	46 (20.0)
Hinduism	42 (18.3)
Others	10 (4.3)
Income per month	
<RM1000 ($250)	82 (35.7)
RM1001-RM2000 ($251–$500)	63 (27.4)
RM2001-RM3000 ($501–$750)	36 (15.7)
RM3001-RM4000 ($751–$1000)	23 (10.0)
RM4001-RM5000 ($1001–$1250)	13 (5.7)
>RM5000 ($1250)	13 (5.7)
Current living conditions^a^	
With spouse	154 (67.0)
With their children	149 (64.8)
Alone	14 (6.1)
With siblings	8 (3.5)
With friends	2 (0.9)
In a nursing home	2 (0.9)
Self-rated health status	
Excellent	2 (0.9)
Very good	14 (6.1)
Good	190 (82.6)
Poor	24 (10.4)
Presence of co-morbidities	206 (89.6)
Hypertension	161 (70.0)
Diabetes	98 (42.6)
Dyslipidaemia	28 (12.2)

^a^more than one answer may be selected; $ = US dollars

### Validity

Factor analysis showed a single dimension among the 5 items in the “feelings regarding ACP” domain (Table 3).

**Table 3:** Factor analysis of the Advance Care Planning Questionnaire

Original domain (no. of items)	New domains (no. of items)	Item	Factor loadings				Average variance extracted	Composite reliability	Keiser-Meir-Olkin
			1	2	3	4			
Feelings regarding advance care planning (5) (*n* = 230)	Feelings regarding advance care planning (5)	Felt better to have expressed wishes in advance if I had a heart attack or on a breathing machine	0.972				0.915	0.999	0.889
		Felt better to have expressed wishes in advance if I had severe dementia	0.961						
		Felt better to have expressed wishes in advance if I had a stroke	0.959						
		Felt better to have expressed wishes in advance if I had cancer	0.952						
		Felt better to have expressed wishes in advance if I had a road accident or in a coma	0.943						
Justifications for advance care planning (7) (*n* = 179)	Justifications for advance care planning (4)	I hope to not burden my family with my medical treatment preferences		0.804			0.597	0.978	0.751
		I want to be able to make my own decision		0.797					
		I am aware that I could possibly lose my decision making power as a result of becoming seriously ill or injured		0.773					
		There may be differences in opinions between my family members		0.713					
Justifications for not having advance care planning (10) (*n* = 55)	Fate and religion (4)	I believed that planning for my death would mean there is no hope for me			0.846		0.624	0.895	0.723
		I believed that the discussion of the topic of death was seen as “unlucky” and I tried to avoid discussing about it			0.830				
		I will take it as it comes, as I have no control over my death			0.759				
		I felt that it was best to leave to fate or to God			0.733				
	Avoid thinking about death (3)	I do not want to think I will die or lose my memory				0.894	0.607	0.840	0.547
		I cannot imagine myself in such a situation				0.850			
		I am currently healthy and there is no need to consider such decisions				0.549			

A total of 179 participants reported that they were in favour of ACP. Factor analysis on the items in the “justifications for ACP” domain gave three underlying domains: “wanting to make my own decision” (4 items), “not trusting others to make a decision for me” (2 items) and “not wanting doctors to perform unnecessary procedures” (1 item). Since the two latter domains only had two and one item(s), respectively, we dropped these 3 items (“An acquaintance has spoken of these issues”, “I do not trust doctors to make the correct decision”, and “When I am gasping for breath, I do not want doctors poking me here and there because of their duty”.

Factor analysis was also performed on 55 participants who were not in favour of ACP. Factor analysis on the items in “justifications for not having ACP” domain gave four underlying domains: “fate and religion” (4 items), “avoid thinking about death” (3 items), “lack of information” (2 items) and “my family will make this decision on my behalf” (1 item). Three items (“I have no information on ACP” and “My doctor will make such decisions when the time comes” in the “lack of information” domain, as well as “My family will make this decision on my behalf”), were omitted from the main construct through factor analysis. However, 74.5 % of the 55 participants answered that they did not have any information regarding ACP, and there was a significant association between “I have no information on ACP” and “My doctor will make such decisions when the time comes”. Similarly, 87.3 % of the 55 participants who were not in favour of ACP, answered that they would let their family make the decision regarding their end-of-life care. Therefore, these items were maintained as independent items in our questionnaire.

### Reliability

Of the 230 participants, only 131(57 %) were available at retest, as the remaining 99(43 %) participants were uncontactable. Reliability analysis was performed on the remaining 17 items. The Cronbach’s alpha values for each domain ranged from 0.637–0.915 (Table 4). All items had corrected item total correlation of >0.2. The deletion of item “I am currently healthy and there is no need to consider such decisions” would increase the Cronbach value from 0.637–0.791. However, this item was retained, as the deletion of this item would result in this domain having only two items, which is unacceptable. In the test-retest reliability analysis, kappa values ranged from 0.738–0.947.

**Table 4:** Reliability of the Advance Care Planning Questionnaire

Original domain (no. of items)	New domains (no. of items)	Item	Cronbach alpha	Corrected item total correlation	Cronbach alpha if item deleted	Cohen’s kappa* (*n* = 131)
Feelings regarding advance care planning (5) (*n* = 230)	Feelings regarding advance care planning (5)	Felt better to have expressed wishes in advance if I had a heart attack or on a breathing machine	0.915	0.935	0.971	0.880
		Felt better to have expressed wishes in advance if I had severe dementia		0.911	0.975	0.814
		Felt better to have expressed wishes in advance if I had a stroke		0.955	0.968	0.860
		Felt better to have expressed wishes in advance if I had cancer		0.925	0.973	0.822
		Felt better to have expressed wishes in advance if I had a road accident or in a coma		0.939	0.971	0.814
Justifications for advance care planning (7) (*n* = 179)	Justifications for advance care planning (4)	I hope to not burden my family with my medical treatment preferences	0.769	0.606	0.699	0.864
		I want to be able to make my own decision		0.594	0.702	0.892
		I am aware that I could possibly lose my decision making power as a result of becoming seriously ill or injured		0.582	0.708	0.769
		There may be differences in opinions between my family members		0.514	0.746	0.802
Justifications for not having advance care planning (10) (*n* = 55)	Fate and religion (4)	I believed that planning for my death would mean there is no hope for me	0.802	0.696	0.713	0.947
		I believed that the discussion of the topic of death was seen as “unlucky” and I tried to avoid discussing about it		0.678	0.720	0.741
		I will take it as it comes, as I have no control over my death		0.572	0.774	0.742
		I felt that it was best to leave to fate or to God		0.540	0.788	0.888
	Avoid thinking about death (3)	I do not want to think I will die or lose my memory	0.637	0.597	0.320	0.770
		I cannot imagine myself in such a situation		0.508	0.478	0.738
		I am currently healthy and there is no need to consider such decisions		0.287	0.791	0.939

*all values were statistically significant at *p* < 0.001

## Discussion

The ACPQ was designed to assess older people’s awareness and acceptance of ACP. It was developed using a systematic and rigorous process according to standard guidelines for developing questionnaires [21]. The final version of the ACPQ consists of 4 sections and 63 items, 30 measured on a nominal scale and 33 on a Likert scale, of which only 19 items were validated. Flesch reading ease was 71. Exploratory factor analysis found that the ACPQ had 4 domains: “feelings regarding ACP”, “justifications for ACP”, “justifications for not having ACP: fate and religion”, and “justifications for not having ACP: avoid thinking about death”. The ACPQ had Cronbach alpha values of >0.6 in each domain and showed adequate reliability.

The ACPQ had a Flesch reading ease of 71. This means that the ACPQ can be read by students aged 12–13 years who are in the 7^th^ grade in the United States [22]. The cognitive debriefing from the pilot study demonstrated that our interviewer-administered questionnaire was easy to understand, once the “medical terms” in the questionnaire were explained to them. Our findings were as expected as previous studies also found that it was easier to administer such questionnaires when it was not self-administered [4, 13, 23]. It was important to assess our instrument for readability even though it was an interview-administered questionnaire, as the interviewer read each item word-by-word from the ACPQ to the participant to ensure consistency of administration.

Exploratory factor analysis showed that the ACPQ had 4 domains: “feelings regarding ACP”, “justifications for ACP”, “justifications for not having ACP: fate and religion”, and “justifications for not having ACP: avoid thinking about death”. As hypothesized, the “feelings regarding ACP” domain only had 1 factor loading, which consists of 5 items that assessed the feelings of participants towards ACP.

In the “justifications for ACP” domain, we found three underlying domains: “wanting to make my own decision (4 items), “not trusting others to make a decision for me” (2 items), and “not wanting doctors to perform unnecessary procedures” (1 item). We retained the first domain: “wanting to make my own decision” (4 items) as it satisfied the AVE, CR and KMO values of a good construct (Table 3). However, the items in the latter two domains were dropped from our instrument for the following reasons. The item “An acquaintance has spoken of these issues” was not a major influencer, as awareness regarding ACP in Malaysia may not be as high as the awareness in developed countries [24]. The item “I do not trust doctors to make the correct decision” did not fit into the first factor loading, as Asians generally prefer doctors to make decisions for them [25, 26]. This is in contrast to patients in developed countries where they would want a more autonomous role in making decisions regarding end-of-life care [27]. Similarly, the item “When I am gasping for breath, I do not want doctors poking me here and there because of their duty” did not perform well. Whilst patients prefer to avoid being in such a situation, Asians generally think that doctors will know what to do in times of medical crisis [25].

Factor analysis on the “justifications for not having ACP” domain revealed two underlying domains: “fate and religion” and “avoid thinking about death”. In previous studies conducted in Malaysia [14] and Singapore [4], participants stated that their religious belief was why they did not want to have ACP, as they felt that it was best to leave their fate to God [4]. This may be because Muslims believe in the afterlife and the Day of Judgment; and that they regard death as a transition from one phase of existence to the next. They believe that illness and disease is a test from Allah, which should be received with faith, patience and prayer [28]. The Chinese elderly in Hong Kong also believed that death is predetermined by destiny which is beyond their control. Hence, participants in this study did not see the point of planning for end-of-life care [29]. In contrast, the majority of Singaporean Chinese nursing home residents reported that they would choose resuscitation, artificial ventilation and nasogastric feeding even in the face of futility, as they feared death and dying, and had a very strong will to carry on living no matter what the circumstances might be [30]. Participants in the Japanese study did not mention about leaving their fate to God [12]. This shows that the willingness to consider future illness and end-of-life care is not universal. As for the domain on “avoid thinking about death”, some communities are known to be reluctant to speak about end-of-life care, as they are “taboo” subjects [31]. In Hong Kong, one qualitative study reported that several Chinese frail old age home residents were reluctant to talk about death as they were afraid of thinking about death and dying issues, and therefore did not want to contemplate the difficult end-of-life care decision [29].

Information is important for decision making. Among those who justified for not wanting ACP, 89 % stated that they were not given enough information to make an informed choice. As a result, many patients expect the health care provider to initiate the discussion regarding ACP [32]. Hence, if doctors were to provide sufficient information to patients regarding ACP, this would facilitate their acceptance of ACP, and complete an advance directive. From the doctors’ perspective, the barriers to initiating a conversation regarding ACP include time constraint, not being adequately trained, and lack of agreement as to whose responsibility is it to initiate ACP discussions and in which setting [6, 33] Although these items (“my family will make this decision on my behalf”, “my doctor will make such decisions when the time is needed”, and “I have no information about ACP”) did not fit well into the main construct, we decided to retain these items as independent items, as these items would be able to explore the extent of the lack of knowledge regarding ACP, as well the individual’s expectation of their doctor to make end-of-life care decisions. Similarly, “My family will make this decision on my behalf”, was retained as an independent item, as 87.3 % of the participants in our study preferred a “family-based decision making process” in regard to their end-of-life care. This may be due to the collectivist society that exists in Malaysia, which reverses the role of the individual, and places more importance within a family to make such major decisions [34]. However, ACP can only occur if the patient has autonomy. In non-maleficence, the family may prefer to protect the patient from the emotional and physical harm caused by directly communicating bad news and addressing death and end-of-life care. In beneficence, the family prefers to encourage the patient’s hope even in the face of terminal illness, and in this situation may not be for ACP [31].

The Cronbach’s alpha values for each domain ranged from 0.637–0.915, indicating that the items in the ACPQ have adequate internal consistency. In test-retest, the kappa values ranged from 0.738–0.947. This indicates that the ACPQ is a reliable instrument.

Ethnicity, religion, and cultural values play an important role in ACP [24, 35, 36]. Malaysia, located in the Western Pacific Region of Asia, is home to Malays (67.4 %), Chinese (24.7 %), Indians (7.3 %) and other Asian ethnic groups (0.6 %) [37]. According to the 2010 Malaysia Population and Housing Census, 61.3 % of the population practices Islam; 19.8 % Buddhism; 9.2 % Christianity; 6.3 % Hinduism; and 1.3 % traditional Chinese religions (such as Confucianism or Taoism). In the Malays (who are predominantly Muslim), death is believed to have its set times for every human being and is expected to come at any time. Death for Muslims is part of the process one has to go through until the Day of Judgment in the afterlife [28]. Chinese families however, would make every attempt to prevent someone from dying and may prefer a relative to stay in the hospital so that resuscitation is attempted at the last minute (usually because they do not want to feel guilty at not doing enough for the loved one during the end-of-life period) [31, 38]. In Christianity, there is belief in resurrection [39]. In Hinduism, there is belief in karma and rebirth [40]; in Buddhism there is belief in the “eight fold path” for Moksha [41]. The benefits of having a validated ACPQ is that researchers would be able to administer this questionnaire to determine if there is any difference in perception among the different ethnic/religious groups in Malaysia. This will then assist policy makers to decide if ACP should be legislated in Malaysia, thus improving end-of-life care in Malaysia.

The strength of this study was that the ACPQ was developed based on Asian studies, one in Japan and the other in Malaysia. However, a limitation of our study was that we could not satisfy the criteria of 10 participants per item for the “justification for not having ACP” domain. Hence, this may potentially limit the strength of our factor analysis. We were not able to perform discriminative validity (in a population that is generally unaware ACP) and convergent validity (as no other ACPQ has been validated in Malaysia). In addition, Flesch reading ease is usually calculated for native English speakers. However, since the ACPQ only requires primary school reading ability, we postulate that it should also be easily understood by those who have completed secondary school education where English is taught as a second language in public schools.

## Conclusions

The English version of the ACPQ consists of 63 items and 4 domains. It was found to be a valid and reliable instrument in assessing the awareness and attitude of older people in Malaysia towards ACP. We intend to administer the ACPQ in a larger cohort of older people in Malaysia, so that the data gathered would provide policymakers with the information required to decide if ACP should be legislated in Malaysia.

## Additional file

Additional file 1: Advance Care Planning Questionnaire. (DOCX 56 kb)

## Abbreviations

ACP: Advance care planning
ACPQ: Advance care planning questionnaire
AVE: average variance extracted
CR: composite reliability
KMO: Keiser-Meir-Olkin
