# Supplementary material for: The development and validation of the advance care planning questionnaire in Malaysia
Source: BMC Med Ethics. 2016 Oct 18;17:61. doi: 10.1186/s12910-016-0147-8 (PMC5069889; doi:10.1186/s12910-016-0147-8)
Supplement: Additional file 1: — Advance Care Planning Questionnaire. (DOCX 56 kb) [file 12910_2016_147_MOESM1_ESM.docx]

**Advance Care Planning Questionnaire**

Thank you for taking the time to complete this questionnaire. This questionnaire will be asking you on your awareness and attitude regarding advance care planning. Advance care planning is a discussion of your medical preferences for a time when you will not be able to make your own healthcare decisions.

Please fill in the appropriate boxes with a tick. Your answers will be kept strictly **confidential**.

| Section A: Demographic information |
| --- |

Please mark the appropriate box or write your response in the space provided.

| A1. | Sex: | Male  Female |
| --- | --- | --- |
| A2. | Age: | ______ years |
| A3. | Marital status: | Single  Married  Divorced  Widowed |
| A4. | Ethnicity: | Malay  Chinese  Indian  Others:________________ |
| A5. | Education: | No formal education  Primary school  Secondary school  Tertiary education |
| A6. | Occupation: | Self employed  Working part time  Working fulltime  Unemployed  Retired/pensioner  *( please state your previous occupation)* : __________ |
| A7. | Religion: | Islam  Hindu  Buddhism  Christianity  Others: ______________ |
| A8. | How much do you earn in a month | <RM 1000  RM 1001 – RM 2000  RM 2001 – RM 3000  RM 3001 – RM 4000  RM 4001 – RM 5000  >RM 5000 |
| A9. | Who do you live with? | No one. I live alone  Spouse  Family members (e.g son, daughter, in-laws)  Siblings  Friends  Nursing home |

| Section B: Health information |
| --- |

| B1. Self-rated health status: | Excellent Very good Good Poor Very poor     \|  \|  \|  \|  \| \| --- \| --- \| --- \| --- \| |
| --- | --- | --- | --- | --- | --- |
| B2. Current medical illness: | No past medical illness  Diabetes mellitus / high blood glucose  Stroke  Kidney problem (on dialysis)  Hypertension / high blood pressure  Heart attack (Myocardial infarction)  Asthma / obstructive airway disease  Liver disease  Cancer  Others:__________ |

| Section C: Awareness on terminologies on advance care planning |
| --- |

| C1. Have you ever heard about advance care planning? |  | Yes |  | No |
| --- | --- | --- | --- | --- |
| C2. Do you know what is advance care planning? |  | Yes |  | No |

| Have you ever heard about the following terminologies related to advance care planning? |  |
| --- | --- |
| C3. Proxy decision maker | Yes  Yes, but I am unsure of its meaning  No (please go to section D) |
| C4. End-of-life decision making | Yes  Yes, but I am unsure of its meaning  No (please go to section D) |
| C5. Living will | Yes  Yes, but I am unsure of its meaning  No (please go to section D) |
| C6. Power of attorney | Yes  Yes, but I am unsure of its meaning  No (please go to section D) |
| C7. How did you hear about the “terms” in C3-C6? | Informed by family doctors  Mass media (Newspapers & TV)  Relatives  Friends  Other sources: (Specify)__________ |
| C8. Have you ever thought of writing a living will?  C9. Have you written one? | Yes  No    Yes  No |

| Section D: Awareness and attitude on advance care planning |
| --- |

| D1. Do you think advance care planning services should be available in your clinic? |  | Yes |  | No |
| --- | --- | --- | --- | --- |
| D2. Do you feel that the discussion on advance care planning would be necessary? |  | Yes |  | No |

We would like to ask you regarding your **previous experience** on the following in the last five years:

| D3. Have you been hospitalised? | Yes |  |  | No |
| --- | --- | --- | --- | --- |
| D4. Have you been involved in the decision making of your treatment? | Yes |  |  | No |
| D5. Do you have any experience involving the care of your family member / relative / friend(s) in the hospital? | Yes |  |  | No |
| D6. Do you have any experience of death of a family member, relative or friend(s)? | Yes |  |  | No |
| D7. Please indicate if you, family member(s) or friend(s) have any experiences with life sustaining treatment (cardiopulmonary resuscitation, mechanical ventilation) | Yes |  |  | No |

In this section, the questions are about your **feelings about advance care planning**. Would you **feel it is better** to have expressed your wishes in advance if:

|  | Strongly  agree | Agree | Do not know | Disagree | Strongly disagree |
| --- | --- | --- | --- | --- | --- |
| D8. You had a stroke |  |  |  |  |  |
| D9. Had a road accident and are in a coma |  |  |  |  |  |
| D10. Had a heart attack and are on a breathing machine |  |  |  |  |  |
| D1. If you were to have a cancer |  |  |  |  |  |
| D12. If you have severe dementia |  |  |  |  |  |

*For those of who have selected **agree or strongly agree,** please answer items D13-D17.

For those that have selected **do not know, disagree or strongly disagree,** please skip this section and answer items D18-27.

| **Items D13-D17**  Please indicate the **reasons** why you feel it is **better** to have expressed your wishes when you are incapable to make a medical decision. | Strongly agree | Agree | Do not know | Disagree | Strongly disagree |
| --- | --- | --- | --- | --- | --- |
| D13. I want to be able to make my own decision |  |  |  |  |  |
| D14. There may be differences in opinions between my family members |  |  |  |  |  |
| D15. I hope to not burden my family with my medical treatment preferences |  |  |  |  |  |
| D16.I am aware that I could possibly lose my decision making power as a result of becoming seriously ill or injured |  |  |  |  |  |
| D17. When I am gasping for breath, I don’t want doctors pocking me here and there because of their duty |  |  |  |  |  |

| **Items D18-D27**  Please indicate the reasons why you feel it is **not better** to express your wishes when you are incapable to make a decision. | Strongly  agree | Agree | Do not know | Disagree | Strongly disagree |
| --- | --- | --- | --- | --- | --- |
| D18. I am currently healthy and there is no need to consider such decisions |  |  |  |  |  |
| D19. I will take it as it comes, as I have no control over my death |  |  |  |  |  |
| D20. I cannot imagine myself in such a situation |  |  |  |  |  |
| D21. I do not want to think that I will eventually die or lose my memory |  |  |  |  |  |
| D22. I felt that it was best to leave my future to fate or God |  |  |  |  |  |
| D23. I believed that planning of my death would mean that there is no hope for me |  |  |  |  |  |
| D24. I believed that the discussion on the topic of death was seen as “unlucky” and I tried to avoid discussing about it. |  |  |  |  |  |
| D25. My family will make this decision on my behalf* |  |  |  |  |  |
| D26. My doctor will make such decisions when the time is needed* |  |  |  |  |  |
| D27. I have no information about Advance Care Planning* |  |  |  |  |  |

*independent items that are not part of the main construct

The following sections will be asking about your **intention to plan your care in advance**

| D28. Has anyone encouraged you to get involved in medical decision if ever you became ill | Yes | No |
| --- | --- | --- |
| D29. Would you consider to discuss on advance care planning in the future | Yes | No |

For those who have chosen “**Yes”,** proceed to answer the following questions. If you have chosen **“No”,** you have completed this questionnaire, thank you.

| Please indicate **what you would like to discuss** your medical preferences if you are incapable to make a decision? | Strongly agree | Agree | Do not know | Disagree | Strongly disagree |
| --- | --- | --- | --- | --- | --- |
| D30. Cardiopulmonary resuscitation (CPR) decision |  |  |  |  |  |
| D31. Artificial breathing / ventilation machine |  |  |  |  |  |
| D32. Tube feeding for nutrition support |  |  |  |  |  |
| D33. Intravenous drip (IVD) |  |  |  |  |  |
| D34. Blood taking |  |  |  |  |  |
| D35. Antibiotics |  |  |  |  |  |
| D36. Place of death |  |  |  |  |  |
| D37. Haemodialysis |  |  |  |  |  |
| D38. Place of care (nursing home / hospital) |  |  |  |  |  |
| D39. Chemotherapy |  |  |  |  |  |

| Please indicate how you would like to **record your advance care planning**? | Strongly agree | Agree | Do not know | Disagree | Strongly disagree |
| --- | --- | --- | --- | --- | --- |
| D40. Written documentation and to give the copy to my health care provider and my family |  |  |  |  |  |
| D41. Verbally to a family member or acquaintance |  |  |  |  |  |
| D42. To make an audio or video tape |  |  |  |  |  |
| D43. Do not know |  |  |  |  |  |

D44. Who would you like to appoint as your decision maker later in an event when you are unable to speak for yourself? Please tick (*√)* one only.

Health care provider

Family members (son, daughter, cousins)

Spouse

Close friends

**Thank you for taking the time to complete this questionnaire.**
